# Management of a crochet hook penetrating perineal injury: A case report

**DOI:** 10.1016/j.eucr.2025.103336

**Published:** 2025-12-30

**Authors:** Asmelash Gebresilase Tewelde, Yirgalem Teklebirhan Gebreziher, Hadush Tesfay Negash, Birhane Mekonen Negash

**Affiliations:** a Department of Surgery, School of Medicine, Colleges of Health Sciences, Mekelle University, Ethiopia; b Department of Pediatric Surgery, Ayder Comprehensive Specialized Hospital, Mekelle University, Ethiopia; c School of Medicine, Colleges of Health Sciences, Aksum University, Aksum, Ethiopia

**Keywords:** Crochet hook injury, Perineal, Case report, Management

## Abstract

Removing an embedded barbed hook in the body without causing further tissue damage is a challenge in the emergency department (ED). A crochet hook is one of these barbed hooks.

A 6-year-old male child presented to our hospital after 1 hour of crochet hook needle injury to the perineum. On perineal examination there was a sharp material imbedded in the anterior perineal area on the right side from the medial raphe. His follow-up course was uneventful.

The advance-and-cut method is the most practiced and successful. The choice of management techniques depends on the anatomic location and depth of the hook.

## Highlights

• Removing an embedded barbed hook in the body without causing further tissue damage is a challenge in the emergency department.
• The advance-and-cut method is the most practiced and most successful.
• The choice of management techniques depends on the anatomic location of the injury and the depth of the injury by the crochet hook.

## 1 Introduction

Removing an embedded barbed hook from the body without causing further tissue damage is a challenge in the emergency department (ED). Most literature on this topic focuses on fish hook removal, but crochet hooks, which are also barbed, can cause trauma to various body parts.^1^^,^^2^ Perineal injuries caused by crochet hooks are rare occurrence. Common removal techniques include advance and cut (push-through), string-yank, needle cover and retrograde methods.^3^ The advance and cut (push-through) technique means pushing the hook through the skin and cutting off the barb then remove the un barbed needle which was the most common method of extraction in fishhook needles (56 %).^1^^,^^2^^,^^4^

For crochet hook needle removal, push-through and needle cover techniques are considered options, with the choice depending on the anatomic site and depth of imbedded needle. To our current knowledge there is no study written on crochet hook penetrating perineal injury or its management.

Here we presented a 6-year-old male child diagnosed with a perineal crochet hook foreign body and successfully treated with the advance and cut (push-through) technique of extraction. This manuscript was prepared following the CARE guidelines (https://www.care-statement.org).

## 2 Case presentation

a. Clinical history and physical examination

**Fig. 1:**
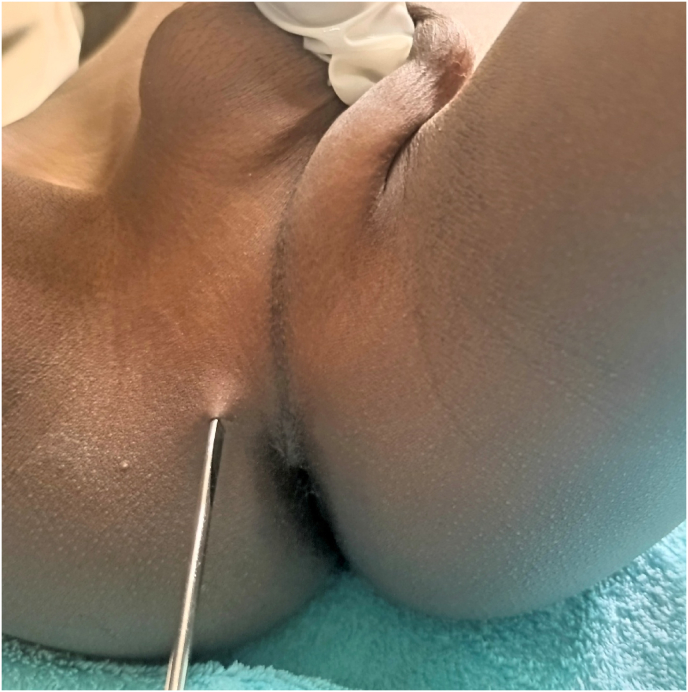
Perineal picture with imbedded foreign body (crochet hook needle) right side of the medial raphe.

This is a 6-year-old male child who presented to our hospital after 1 hour of crochet hook needle injury to the perineum. He accidently sits on the metal. He had a history of perineal pain but no bleeding from the site or in urine. He also had no trauma to other site of the body. On physical examination the vital signs were within the normal limits; on perineal examination there was a sharp material (crochet hook) imbedded on the anterior perineal area on the right side from the medial raphe (Fig. 1), with no blood on the urethral meatus.

b. Diagnostic investigations

**Fig. 2:**
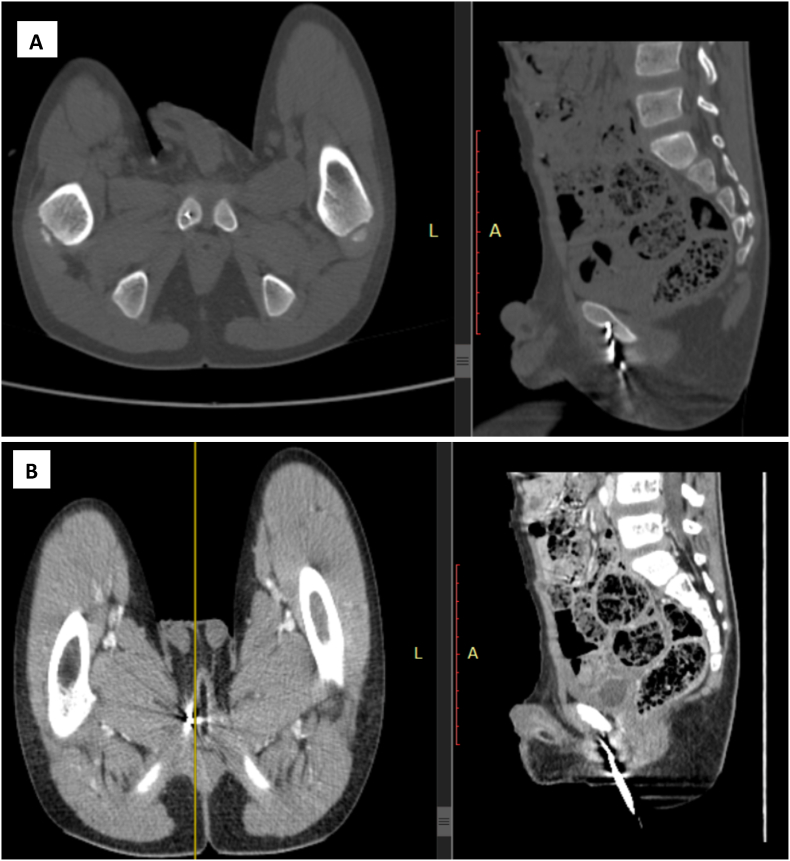
Axial and Sagittal views of Pelvic CT scan, (A) bone window which shows hyperdense lesion in the perineal soft tissue with metallic artifacts. (B) postcontrast view of the same image.

Baseline investigations: leukocyte count was 8800 cells/μL, hemoglobin was 12.5 g/dl and platelet count was 238,000/μL. A pelvic x-ray showed radio-opaque metallic foreign body in the perineum and a pelvic computed tomography (CT) scan with contrast showed radio-dense lesion on the perineal subcutaneous tissue extending up to the outer surface of the right superior pubic ramus (Fig. 2).

c. Treatment and follow-up

**Fig. 3:**
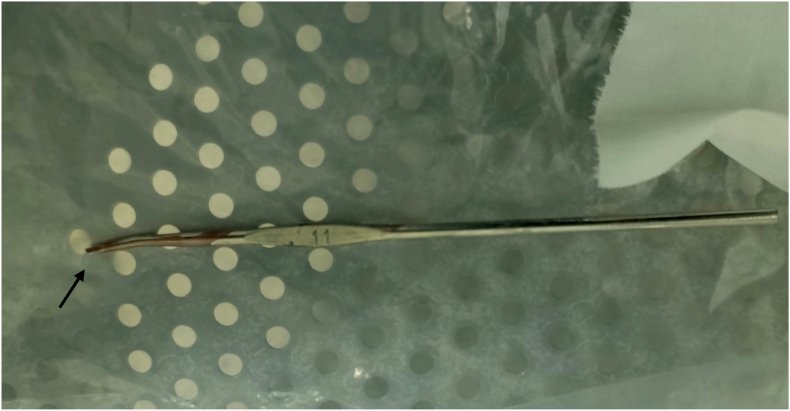
The removed crochet hook needle. The barbed end of the needle is cut (arrow).

With the diagnosis of crochet hook penetrating perineal injury, he was catheterized and prepared for operation. In the major operation room under sedation and local anesthesia, he was positioned on lithotomy position and the perineum was prepared with the standard sterile way and dressed. The crochet hook was detached from the pubic ramus and advanced to the skin. A small incision was done on the skin over the tip of the crochet hook with a number 11 blade and after the barbed part of the needle was exposed, it was cut by a wire cutter and pulled through the way in (advance and cut technique) (Fig. 3). The wound was dressed and the patient was transferred to the post-anesthesia care unit. He was discharged from our hospital on his 2nd postoperative day with two-week appointment to the Pediatric Surgical Referral Clinic (P-SRC). On P-SRC follow-up, he had no new complaint and was discharged from the hospital.

## 3 Discussion

The history and physical examination are crucial for assessing the type of trauma and evaluating injuries both inside and outside the perineum.^2^^,^^5^ Baseline investigation and imaging's also help with management of foreign bodies and the extent of depth. In our case pelvic CT scan was used to locate the position of the crochet hook and its depth in 3D view, because it is difficult to comment the extent of depth and location on plain pelvic x-ray.

There is a detailed description of four methods for removing hooked or barbed foreign bodies.^2^^,^^6^, ^7^, ^8^, ^9^ They were all designed to lessen the possibility that the barb might sever more tissue during retrograde movement and result in additional damage. The nature of fishhook, location of the damage, and the depth of tissue penetration are all factors in the removal technique that is selected.^6^^,^^8^ The most practiced and most successful, especially in fish hooks, is the advance and cut method.^4^^,^^8^ We use this technique of removal because the location and depth of the hook was superficial not involving deep pelvic organs. in one case series there was advance and without cut procedure, were the barb is advanced through the skin and the hooked part is bent to the main shaft.^3^ This method can be used if wire cutter is not available.

The advance and cut method: The embedded hook is advanced through the skin using this approach, as the name suggests, so that the barb can be snipped off and the rest of the hook can be removed. The barb is removed once it has passed through the anesthetized skin. A retrograde direction can then be used to remove the hook.^6^^,^^7^ This method was successful in 56 of 97 cases where this was studied (58 %).^8^ When the hook cannot be advanced to cut, as is frequently the case in the finger when bone or fingernail is involved, this approach is limited.^6^

Because the retrograde and string-yank methods generate the least amount of tissue trauma, they should be frequently tried first.^9^ Despite being the most straightforward removal method, the retrograde procedure has the lowest success rate. When removing hooks that are situated very superficially, this could work effectively.^6^^,^^9^ The string-yank method cannot be applied for crochet hook removal due to the nature of the needle. By trapping the hook inside the hollow point of a needle, the needle cover method is intended to neutralize the hook's barb.^6^ Along the hook's entrance wound, a needle of at least 18 gauge is advanced. It is important to insert in a direction parallel to the shank.^9^ The hook and the needle will be pulled out retrogradely.

There are case reports of crochet hook injury to other body parts; to the oral cavity removed by the needle cover method,^2^ and both orbits removed by the advance and cut method.^10^

## 4 Conclusion

The choice of management techniques depends on the anatomic location of the injury and the depth of the injury by the crochet hook. Advance and cut can be the preferred choice if the anatomic location allows.

## CRediT authorship contribution statement

**Asmelash Gebresilase Tewelde:** Writing – review & editing, Writing – original draft, Visualization, Data curation, Conceptualization. **Yirgalem Teklebirhan Gebreziher:** Writing – review & editing, Writing – original draft, Visualization, Data curation, Conceptualization. **Hadush Tesfay Negash:** Writing – review & editing, Writing – original draft, Visualization, Data curation, Conceptualization. **Birhane Mekonen Negash:** Writing – review & editing, Writing – original draft, Visualization, Data curation, Conceptualization.

## Informed consent

Written informed consent was obtained from the patient's parents for publication of this case report and accompanying images. A copy of the written consent is available for review by the Editor-in-Chief of this journal on request.

## Informed consent

Informed consent for the publication of this case has been obtained from the patient's parents.

## Trial registration number

Not applicable.

## Grant number

Not applicable.

## Ethical statement

This type of study does not require any ethical approval by our institution.

## Date availability statement

The authors confirm that the data supporting the findings of this study are available within the article and its supplementary materials.

## Authorship

All authors attest that they meet the current ICMJE criteria for authorship.

## Funding statement

No funding was received.

## Declaration of interests

The authors declare that they have no known competing financial interests or personal relationships that could have appeared to influence the work reported in this paper.

## Abbreviations

ED -: emergency department
CT -: computed tomography
